# Neurofeedback-dependent influence of the ventral striatum using a working memory paradigm targeting the dorsolateral prefrontal cortex

**DOI:** 10.3389/fnbeh.2023.1014223

**Published:** 2023-02-09

**Authors:** Daniela Jardim Pereira, Alexandre Sayal, João Pereira, Sofia Morais, António Macedo, Bruno Direito, Miguel Castelo-Branco

**Affiliations:** ^1^Neurorradiology Functional Area, Imaging Department, Coimbra Hospital and University Center, Coimbra, Portugal; ^2^Coimbra Institute for Biomedical Imaging and Translational Research (CIBIT), University of Coimbra, Coimbra, Portugal; ^3^Faculty of Medicine, University of Coimbra, Coimbra, Portugal; ^4^Institute of Nuclear Sciences Applied to Health (ICNAS), University of Coimbra, Coimbra, Portugal; ^5^Siemens Healthineers Portugal, Lisboa, Portugal; ^6^Psychiatry Department, Coimbra Hospital and University Center, Coimbra, Portugal; ^7^IATV—Instituto do Ambiente, Tecnologia e Vida (IATV), Coimbra, Portugal

**Keywords:** DLPFC, ventral striatum, neurofeedback, working memory, motivation

## Abstract

Executive functions and motivation have been established as key aspects for neurofeedback success. However, task-specific influence of cognitive strategies is scarcely explored. In this study, we test the ability to modulate the dorsolateral prefrontal cortex, a strong candidate for clinical application of neurofeedback in several disorders with dysexecutive syndrome, and investigate how feedback contributes to better performance in a single session. Participants of both neurofeedback (*n* = 17) and sham-control (*n* = 10) groups were able to modulate DLPFC in most runs (with or without feedback) while performing a working memory imagery task. However, activity in the target area was higher and more sustained in the active group when receiving feedback. Furthermore, we found increased activity in the nucleus accumbens in the active group, compared with a predominantly negative response along the block in participants receiving sham feedback. Moreover, they acknowledged the non-contingency between imagery and feedback, reflecting the impact on motivation. This study reinforces DLPFC as a robust target for neurofeedback clinical implementations and enhances the critical influence of the ventral striatum, both poised to achieve success in the self-regulation of brain activity.

## Introduction

Neurofeedback (NF), as an operant conditioning of brain activity, has been largely performed for different training purposes and in distinct experimental groups—initially with EEG (Lévesque et al., 2006; Kouijzer et al., 2009) and, in the last decades, taking profit of the greater spatial resolution of real-time fMRI (Weiskopf et al., 2003). It has also been applied in several clinical conditions such as depression (Linden et al., 2012; Young et al., 2017; Mehler et al., 2018; Takamura et al., 2020), stroke (Sitaram et al., 2012; Liew et al., 2016), obesity, and overweight (Frank et al., 2012; Spetter et al., 2017; Kohl et al., 2019), neurodegenerative disease (Subramanian et al., 2016; Hohenfeld et al., 2017; Papoutsi et al., 2018), chronic pain (DeCharms et al., 2005; Guan et al., 2015), ADHD (Alegria et al., 2017; Zilverstand et al., 2017; Rubia et al., 2019), ASD (Ramot et al., 2017; Pereira et al., 2019; Direito et al., 2021) and addiction (Martz et al., 2020), with globally encouraging results. However, a deeper understanding of the underlying cognitive strategies and neurofeedback-specific neural networks is still required before fully translating these experimental science efforts into rehabilitation in clinical practice.

A number of key regions have been implicated in cognitive processes related to neurofeedback, these including self-regulation/control, learning, and reward, regardless of the brain areas that are activated by the specific mental task (Emmert et al., 2016; Sitaram et al., 2017; Paret et al., 2019). Three main networks are involved in neurofeedback: (1) the fronto-parietal network (FPN), also termed executive control network, which includes the dorsolateral pre-frontal cortex (DLPFC) and posterior parietal cortex (PPC); (2) the cingulum-opercular network (CON), also known as the salience network, anchored on anterior insula (aI) and anterior cingulate cortex (ACC); and (3) the basal ganglia network (BGN). Thalamus and visual association areas are also involved in neurofeedback processing, through their connection with the aforementioned networks. CON underlies conscious perception of feedback, and, in its interaction with the ventral striatum (from BGN), also takes part in unconscious reward processing. Dorsal striatum (from BGN) has been implied in neurofeedback procedural learning. FPN is well established as a key component of the neurofeedback network, being activated by the mental imagery task itself (Zvyagintsev et al., 2013; Spagna et al., 2021), during strategy execution period, but also during feedback processing along with CON (Dewiputri et al., 2021). Our present work focuses on the core of FPN, the dorsolateral prefrontal cortex (DLPFC).

The dorsolateral prefrontal cortex (DLPFC), a cluster of functional brain regions identified in humans and other primates is a key hub for executive functions (Elliott, 2003; Niendam et al., 2012)—an umbrella term covering multiple high-order cognitive functions, such as working memory, attention, cognitive flexibility, action planning, and inhibition of inappropriate behaviors. Specific functional contributions of DLPFC in executive functions include maintenance of information for goal-directed activity, manipulation, response selection and inhibition (Niendam et al., 2012; Rabinovici et al., 2015). The large set of functional coverage makes impairments in executive functions poor as a disease specific physiopathological model but very attractive as a potential neurorehabilitation target. If we could successfully train FPN in association with positive clinical outcomes, it will have significant day-to-day functional impact in a number of neurological and psychiatric diseases, where executive dysfunction emerges (even if in a non-causal way) traduced in impaired intellectual ability and disrupted adaptive behavior (Enriquez-Geppert et al., 2013; Rabinovici et al., 2015).

Accordingly, the dorsolateral prefrontal cortex (DLPFC) has been used as a neurofeedback training target both in health (Zhang et al., 2013; Sherwood et al., 2016a, b; van den Boom et al., 2018; Travassos et al., 2020; Yu et al., 2021; Weiss et al., 2022) and disease, including depression (Takamura et al., 2020), anxiety (Lisk et al., 2020; Morgenroth et al., 2020), overweight/obesity (Spetter et al., 2017; Kohl et al., 2019) and craving (Karch et al., 2015, 2019). Most of these studies were founded on the role of DLPFC in inhibition of inappropriate behavior and/or top-down control (namely in the interaction with the affective brain circuits), some of them performing connectivity-based neurofeedback (Spetter et al., 2017; Lisk et al., 2020; Morgenroth et al., 2020; Weiss et al., 2022). Here, similarly to studies from Zhang, Sherwood and van den Boom research groups, we target DLPFC because of its pivotal role in working memory.

In this sham-controlled single session study, we validate a rt-fMRI neurofeedback training framework for DLPFC self-modulation using a working memory paradigm on healthy subjects (without executive dysfunction). The paradigm is based on a backward digit-span, a working memory task often used in neuropsychological assessment, adapted to a neurofeedback/imagery task by Zhang et al. (2013). In our study, we now include both train and transfer runs, to evaluate whether the participants are able to enhance the target activation even without feedback and if neurofeedback runs improve this ability. Furthermore, a recent machine learning mega-analysis pointed out that a pre-training no-feedback run was one of the two factors influencing neurofeedback performance (Haugg et al., 2021).

The second critical modification we introduced was the origin of feedback signal in the sham group, which in our case were regions of interest (ROIs) placed in the white matter, as opposed to the yoked feedback used in Zhang et al. (2013) study and van den Boom et al. (2018), and the n-back train without feedback in Sherwood et al. (2016a,b). There is no consensus about the optimal control condition for rt-fMRI NF studies, however feedback from an alternative brain signal seems to control for most of the factors in order to establish causality, namely, it conceptually demonstrates neurophysiological specificity, in contrast with yoked feedback (Sorger et al., 2019). In our working memory paradigm is very challenging to find an independent brain region, considering the broad coverage of the executive network and connectivity with other relevant networks, such as the salience network and the default mode network (DMN), and even subcortical structures and the cerebellum—circuits that studies from Zhang’s group shown further to be modified by their NF paradigm (Shen et al., 2015; Zhang et al., 2016). This was the rationale to choose white matter as control ROI—an independent BOLD signal. A possible drawback is that the lower amplitude (Gawryluk et al., 2014) may derive a higher probability of participants becoming aware of the non-contigency of neurofeedback signal, ultimately influencing motivation.

A final contribution was the investigation of the temporal properties of neurofeedback processing within the activation block by extracting the mean BOLD signal time-courses of the target ROI. We expect this could give us a better insight of possible differences between neurofeedback and sham groups in the immediate response to feedback and consequent dynamical adjustments inside the block.

In this study, we aim to engage the central executive network (CEN), specifically the DLPFC, validating a potentially robust workflow to be implemented in a number of clinical groups where executive dysfunction plays a significant role, with individual, social and economic impact. We test the ability of healthy subjects to modulate this functionally defined target area through mental imagery, using a working memory task. We compare these results with the sham-control group, receiving feedback from a set of white matter voxels evenly distributed in each participant’s *centrum semiovale*, trying to disentangle the functional overlap of DLPFC as a key region for neurofeedback processing, along with other critical networks such as the reward system. Thus, we hypothesize that:

(1) The active NF group would be able to achieve greater control over signal modulation in the DLPFC when compared to the sham-control group, given the correspondence between feedback and mental strategy.
(2) Reward networks (CON and BGN) involved in neurofeedback processing will significantly contribute to this difference in this one single session experiment.

## Material and methods

### Participants

Twenty-seven healthy volunteers participated in this study. Seventeen subjects were allocated to the active neurofeedback group (10 male, mean age 27.8±4.2 years) and 10 subjects to the sham neurofeedback group (5 male, mean age 26.2±2.9 years). All had a normal or corrected-to-normal vision and no history of neurological or psychiatric diseases. All participants except one were right-handed. All gave informed written consent before participating, in accordance with the declaration of Helsinki, and the study complied with the safety guidelines for magnetic resonance imaging (MRI) research on humans. The work was approved by the Ethics Committee of the Faculty of Medicine of the University of Coimbra.

### Experimental protocol

**Figure 1:**
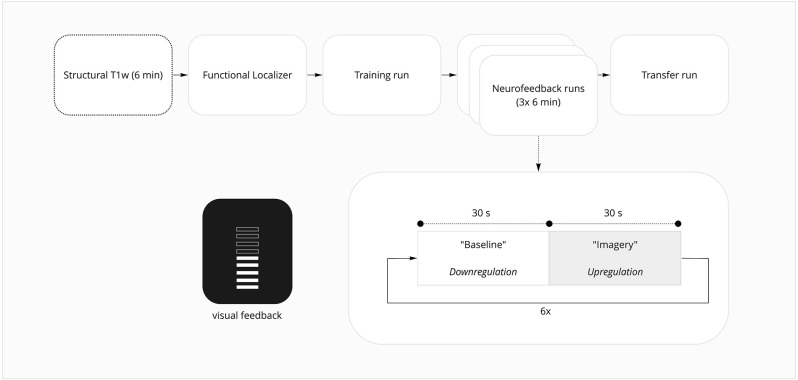
fMRI session overview.

The experimental session was composed of an anatomical run—where structural brain information is acquired—followed by six functional runs—task-related runs where brain activation is inferred by measuring the blood oxygenation level-dependent (BOLD) signal (Figure 1). These runs consisted of a localizer run followed by five imagery runs. The localizer run was used to functionally map the DLPFC spatial mask used in the following runs as the neurofeedback target region. The first and the last imagery runs were performed without providing feedback information to the participant. The scanning session lasted approximately 1.5 h, followed by a debriefing questionnaire.

#### Functional localizer

The localizer task consisted of three conditions: a 1-back and a 2-back condition distributed randomly in 10 blocks (five blocks per condition), alternating with baseline blocks. Each block has 15 volumes and 15 digits, with five of them being targets and the remaining 10 non-targets. Each digit was displayed for 400 ms. Each block was preceded by the instruction to remember one or two preceding numbers. This stimulus was created and presented in Presentation^®^ software (Version 20.1, Neurobehavioral Systems, Inc., Berkeley, CA, www.neurobs.com).

Participants were instructed to press a button when the number displayed matches the one from one step earlier in the sequence (1-back condition) or two steps earlier (2-back condition; specific task instructions to participants are described in detail in Supplementary Material). The total length of the run was 10.5 min, and participant responses were recorded through an MR-compatible response box (Cedrus Lumina LSC-400B).

For online ROI definition, we functionally targeted DLPFC using the real-time fMRI software package Turbo-BrainVoyager 3.2 (TBV; Brain Innovation, Maastricht, The Netherlands). Real-time preprocessing included 3D head motion correction (6 degrees of freedom) compared to the first volume. Online statistical analysis of incoming volumes was incremental, using a recursive least squares general linear model (GLM) based on a design matrix automatically created from the imported stimulation protocol and including the convolution of the BOLD time course with a two-gamma hemodynamic response curve (HRF).

Activation clusters were estimated, in a first approach, according to the contrast “2-back” > “baseline” that usually resulted in the highest percent of signal change (PSC). However, in some participants, we found very large clusters of activation in DLPFC (merging with pre-motor areas) with this contrast. In these cases, switching to the “2-back” > “1-back” contrast allowed us to delineate a more circumscribed effort-related cluster, associated with cognitive-load and manipulation, and find a more accurate peak voxel to center the rectangle drawn on multi-slice view of TBV. The defined rectangle extended to the slice above and below (a total of three slices) and the average of significant voxels was displayed in the time-course. We generally considered ROIs appropriate as NF targets, when their PSC was around 1% or more. Anatomical references were also taken into account by an expert neuroradiologist (DP) to determine DLPFC, guaranteeing it was located anterior to the premotor cortex and superior to the planes including the lateral ventricles. All targets were selected on the left hemisphere since participants were performing a verbal working memory task during imagery runs (Emch et al., 2019).

For the sham feedback group, we selected between 18 and 24 functional voxels of white matter, evenly distributed per hemisphere, in each participant *centrum semiovale*.

#### Imagery runs

The imagery runs included two conditions—“imagery” and “baseline”—presented alternatively six times per run with an additional “baseline” block at the beginning of each run, each condition block lasting 30 s. During the neurofeedback runs, participants were instructed to empty the thermometer during “baseline” conditions and increase the thermometer bars during the “imagery” condition. Subjects were instructed to use a cognitive strategy of backward reciting the self-generated sequences sub-vocally to increase the number of bars in the thermometer (Zhang et al., 2013). The content, length, and difficulty of the sequences they generated and the speed of recitation could be adjusted according to the feedback. Instructions provided to the neurofeedback and sham groups were identical and none of the participants was aware of the existence of a control condition. For detailed instructions, as provided to participants, please refer to Supplementary Material.

#### Debriefing

After the scanning session, participants answered a debriefing questionnaire that included subjective questions about their feelings during the acquisition (*How did you feel during the NF session?*), the contingency between effort and feedback change (*Did you feel there was a correspondence between the used strategies and the given feedback?)* and the strategies they used (*What was the maximum number of sequences you could picture in each block? And the maximum digit number? Which strategies worked better? And which ones did not work?*). An independent sample *t*-test was performed to evaluate group differences in the number of digits and sequences generated by the participants.

### Data acquisition

MRI data acquisition was conducted on a 3T Siemens Magnetom TrioTim scanner with a 12-channel head coil. For the anatomical runs we used a high-resolution magnetization-prepared rapid acquisition gradient echo (MPRAGE) sequence (176 slices; echo time (TE): 3.42 ms; repetition time (TR): 2,530 ms; voxel size: 1 × 1 × 1 mm; flip angle (FA): 7°; field of view (FOV): 256 × 256 mm). Functional imaging was acquired with an echo-planar imaging (EPI) sequence with 32 slices, in-plane resolution: 3 × 3 mm, FOV: 192 × 210 mm, matrix 64 × 70, slice thickness: 2.5 mm, FA: 75°, TR = 2,000 ms and TE = 30 ms. Functional runs included two “dummy scans” at the beginning of the acquisition (discarded and not stored) that allowed the magnetization to stabilize to a steady state.

### Feedback calculation and presentation

During the neurofeedback runs, apart from the first baseline block, visual feedback was provided in the form of a thermometer that was updated every TR based on the mean ROI activation of the neurofeedback target selected during the localizer run. In order to correct for head movements across runs, we performed an intra-session alignment (six degrees of freedom), using the first volume of the localizer as a reference for all runs.

The thermometer was divided into 10 discrete levels with a maximum value of 2.5%, where each level represented a given range of percent BOLD signal change (0 for an empty thermometer and 0.25% for each level). The feedback value *fb* for the current time point *n* is calculated within each block given the current value *val*, a baseline level *bl* (mean BOLD value in the target region, during the previous “baseline” block) according to equation 1:


(1) $$fb\left(n\right)=\frac{val\left(n\right)-bl}{bl}\times 100$$

### fMRI data analysis

Offline fMRI data analysis was performed using BrainVoyager QX 2.8 (Brain Innovation, Maastricht, The Netherlands). Preprocessing steps included slice scan time correction, 3D motion correction (6 degrees of freedom), temporal high-pass filtering (GLM Fourier method, two cycles, i.e., a GLM with predictors that accommodate sine and cosine functions with two cycles over the entire time-course of the run), spatial smoothing using a 3D gaussian kernel (FWHM = 6 mm), and normalization to Talairach (TAL) coordinate space.

First-level analysis was performed using a GLM for each run. The design matrix included a predictor for each experimental condition convolved with the BrainVoyager’s default two-gamma HRF, and confound predictors for the six motion parameters (three translational and three rotational) and motion spikes (volumes with a root mean square displacement greater than 0.25).

Second-level analysis was based on a random effects (RFX) GLM, correcting for multiple comparisons with false discovery rate (FDR; *q* = 0.005).

#### Localizer for DLPFC

A group activation map was generated for the localizer run combining participants from the active and sham groups, using RFX-GLM contrasting the “2-back” and “baseline” conditions (FDR-corrected *q* = 0.005).

#### Characterization of the target region

To characterize the target ROIs selected online for each participant, we computed the *t*-value for the contrast of interest (“2-back” > “baseline”) during the localizer run and estimated the size (number of voxels) and the center coordinates of each ROI. To measure the variability of ROI definition across participants from the active group, we created a probability map of the overlap (%) between the subject-specific ROIs.

#### DLPFC modulation across subject groups

First, we computed the *t*-value in DLPFC for the contrast of interest (“imagery” > “baseline”) for all imagery runs in both groups. We used this value as the measure of the participant’s ability to modulate the target region.

To assess the effect of group assignment (between-subjects factor, active neurofeedback group vs. sham feedback group) on DLPFC modulation across the five runs (within-subject factor) we performed a mixed-model ANOVA. To analyze if between group differences were determined by neurofeedback and not only related with imagery task performance (closed loop vs. open loop), we performed a two-way ANOVA considering the group assignment as a fixed factor and runs without feedback (average of train and transfer runs) and runs with feedback (average of the three NF runs) as dependent variables.

To evaluate the existence of a within-session learning effect on the active group, we performed a paired sample *t*-test between modulation ability during the transfer and train runs for both groups.

#### Whole-brain analysis

We analyzed whole-brain statistical maps of the NF runs for the active group. Using an RFX-GLM map, we contrasted “imagery” and “baseline” conditions (FDR-corrected *q* = 0.005), revealing the brain regions recruited by this cognitive process. We summarized the center coordinates and *t*-values of each cluster.

Laterality index (LI) was quantitatively assessed using the LI-tool (Wilke and Lidzba, 2007) over the whole brain (excluding the occipital lobe), based on bootstrapped LI curves.

To further explore the mechanisms of contingent feedback, we checked for whole-brain differences between the active and sham groups during the neurofeedback runs. We used a standard repeated-measures ANOVA in BrainVoyager with *condition* (“imagery” and “baseline”) as within-subjects factor and *group* (active vs. sham) assigned as between-subjects factor.

### Event-related average responses

We examined the event-related average (ERA) responses of functionally relevant ROIs extracted from the previous analyses. To this end, the mean BOLD signal time-courses of the DLPFC and ventral striatum were converted into PSC (signal variation relative to the average BOLD value during the “baseline” condition). Then, the time-course was segmented based on the onset and offset of each “Imagery” condition block (we considered two volumes before the onset and five after the end of each block to better understand the temporal profile of the response). Finally, we averaged these segments across trials and participants for each group, allowing for the between group comparison of the response of each brain region.

## Results

### Characterization of the neurofeedback target region (ROI Analysis)

#### Localizer for DLPFC

The definition of the subject-specific target ROI on the DLPFC for both groups was performed based on the online statistical map of the localizer run, contrasting the “2-back” against the “baseline” blocks. Coordinates in Talairach space, number of voxels and *t*-value for the contrast of interest of the selected ROIs for each participant are provided as Supplementary Table 1.

**Figure 2:**
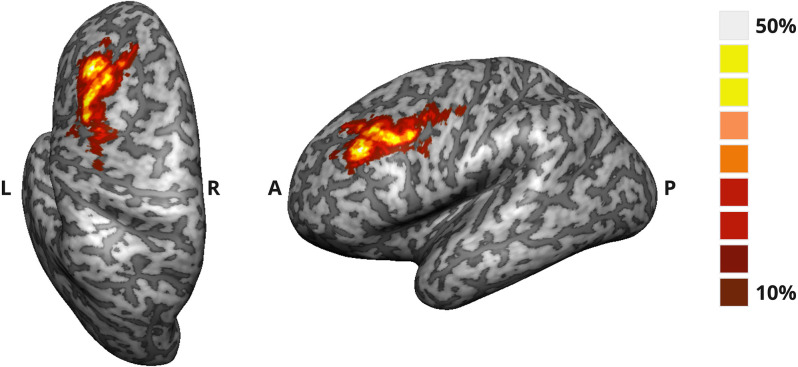
Probability map of the target ROI selected online in the DLPFC for the active NF group. The map translates the degree of overlap (%) between the ROIs defined for each participant.

In Figure 2, we display the probabilistic map for the target ROIs used to provide feedback to the active NF group, from which we can assess the degree of overlap of the ROIs defined online for each participant of this group.

In the sham group, providing feedback from white matter ROIs was variable and presented a positive display (thermometer with at least one level) for almost 40% of the timepoints, although with lower amplitude. Histograms representing the distribution of time points (number of given feedbacks) by thermometer level (1–10) for all blocks (baseline and imagery) in all the three neurofeedback runs are presented as Supplementary Figure S1.

#### DLPFC modulation across subject groups

**Figure 3:**
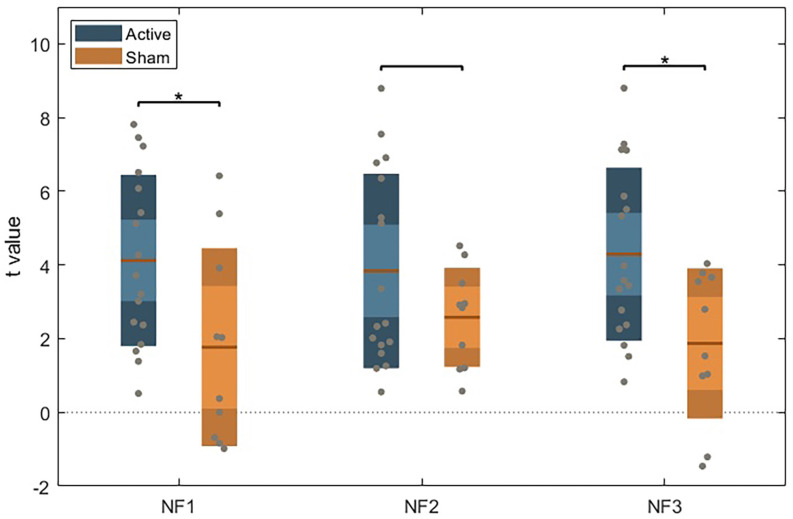
Unpaired *t*-test between the active and sham group for each NF run, using Bonferroni’s correction. Dark color areas represent one standard deviation; light color areas represent the 95% confidence interval for the mean. Gray dots represent data for each subject. The brown line is the mean for each group.

We did not find a significant interaction between group assignment and runs (*F*_(4, 22)_ = 1.617), *p* = 0.205) in the mixed-model ANOVA. However, we found a significant effect for group assignment (between-subject; *F*_(1, 77)_ = 5.056, *p* = 0.034). Within neurofeedback runs, *post-hoc* independent sample *t*-tests (with Bonferroni correction) show significant differences in DLPFC activity between groups in runs 1 (*p* = 0.024) and 3 (*p* = 0.012), as represented in Figure 3.

When comparing runs with feedback and without feedback (train and transfer) to evaluate group differences related to feedback contingency (two-way ANOVA), we found a group assignment effect (*F*_(2, 24)_ = 3.860, *p* = 0.035) with significant difference between groups only in neurofeedback runs (*p* = 0.014) on *post-hoc* pairwise comparisons (with Bonferroni correction).

**Figure 4:**
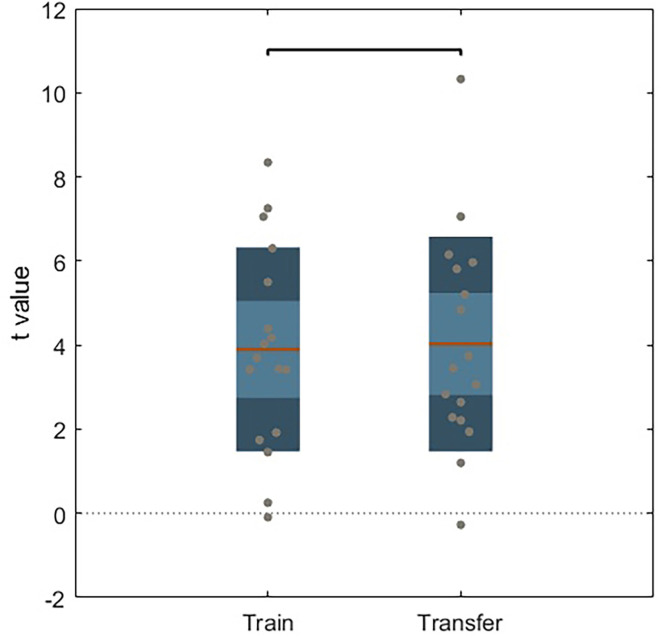
Unpaired *t*-test between train and transfer for active NF group. Dark color areas represent one standard deviation, light color areas represent the 95% confidence interval for the mean. Gray dots represent data for each subject. The brown line is the mean for each group.

No significant differences were found on paired sample *t*-test between train and transfer runs on the active NF group (Figure 4) or in the sham group (no learning effect).

### Exploring brain regions involved in working memory and neurofeedback (whole brain analysis)

#### Localizer and imagery in active group (*N* = 17)

**Figure 5:**
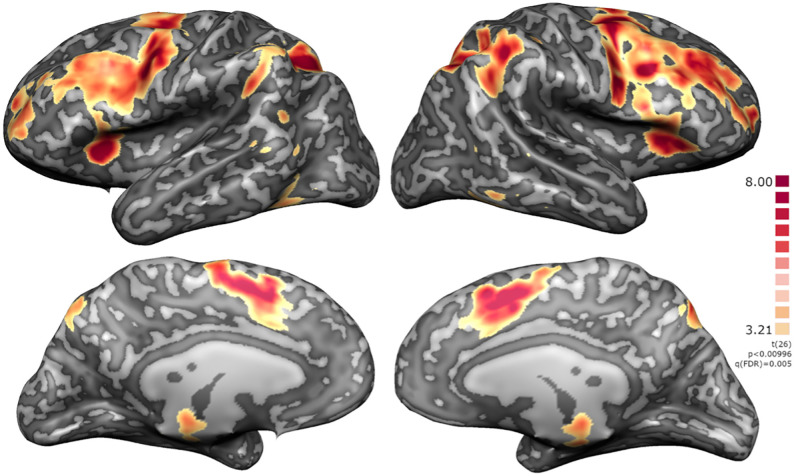
Localizer RFX map for *N* = 27. Activation clusters include regions integrated in executive functions network, namely DLPFC, premotor cortex, supplementary motor area (SMA), basal ganglia, thalamus, intraparietal sulcus (IPS), anterior insula, superior frontal gyrus (SFG), superior vermis (cerebellum), and red nucleus/substantia nigra (RN/SN).

Whole-brain analysis of the localizer run, considering the contrast “baseline” < “2-back”, highlighted clusters on the DLPFC, premotor cortex, supplementary motor area (SMA), basal ganglia, thalamus, intraparietal sulcus (IPS), anterior insula, superior frontal gyrus (SFG), superior vermis (cerebellum), and red nucleus/substantia nigra (RN/SN; Figure 5).

Considering the active group during the neurofeedback runs (baseline < imagery), we identified a significant overlap with the localizer run, except that in the imagery task an evident left lateralization of fronto-parietal activations was also noted. This visual impression was quantitatively assessed and the laterality index confirms a predominantly left-hemispheric activation (LI 0.26, above 0.2 considered as reliable evidence for lateralization).

**Figure 6:**
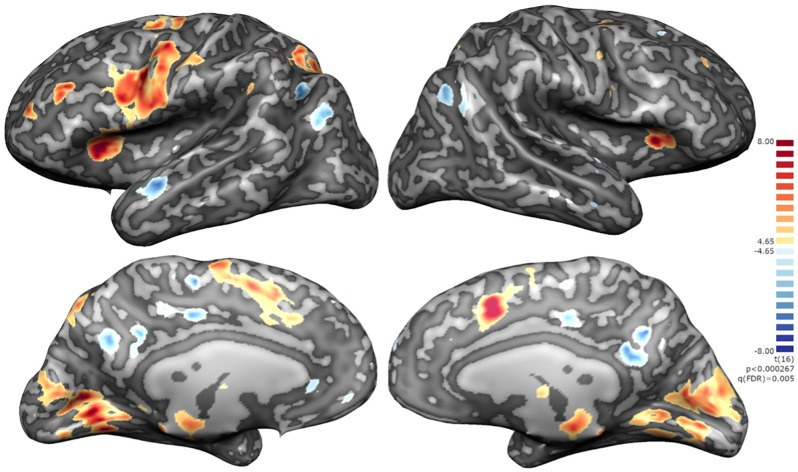
Whole-brain statistical maps of the NF runs for the active group. Positive clusters integrating the executive network with a left lateralization of fronto-parietal activations and negative clusters integrating the default mode network namely mOFC, CCGp and precuneus, middle temporal gyrus (MTG), angular gyrus and parahippocampal cortex [q(FDR) on the neurofeedback runs].

A summary of the activation clusters during neurofeedback runs is provided in Table 1, together with the average of the coordinates in Talairach space and *t*-value for the contrast of interest. Figure 6 displays both positive and negative activation clusters.

**Table 1:** Clusters from Whole-Brain Analysis, active feedback group, imagery > baseline, FDR (*q* = 0.005), min cluster size 20 voxels, MNI coordinates of peak.

**Area (BA)**	**X**	**Y**	**Z**	***t*-value of peak voxel**
Right-AngGyrus (39)	53	−64	28	−7,799,764
Right-Broca-Operc (44)	34	16	15	9,836,548
Right-Insula (13)	41	−12	−3	−7,094,061
Right-PreMot+SuppMot (6)	26	−12	64	937,252
Left-PreMot+SuppMot (6)	−47	5	36	11,785,719
Outside defined BAs	4	56	42	−5,892,329
Left-PreMot+SuppMot (6)	−4	−22	50	−10,484,083
Right-DorsalACC (32)	0	52	6	−7,503,253
Left-AngGyrus (39)	−39	−52	46	9,083,226
Left-dlPFC (dorsal; 9)	−40	28	40	8,301,716
Left-PreMot+SuppMot (6)	−47	5	36	11,785,719
Left-AngGyrus (39)	−55	−63	24	−7,385,214
Left-SupTempGyrus (22)	−57	−5	−10	−7,367,359

#### Active vs. sham

**Figure 7:**
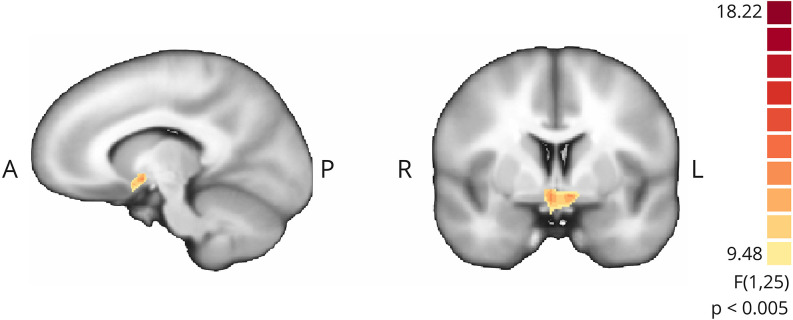
Whole-brain exploratory analysis (mixed ANOVA, *p* < 0.005, non-corrected) between active NF and sham groups. Positive cluster in ventral striatum bilaterally.

To assess the impact of providing contingent feedback, i.e., effect of group assignment (active neurofeedback group vs. sham feedback group), we analyzed the difference of mean modulation between groups, as measured by the contrast of interest (“imagery” > “baseline”), during neurofeedback runs. A cluster located on ventral striatum, apparently in correspondence to nucleus acccumbens, emerged from this exploratory between group comparison (Figure 7).

### Event-related average responses

#### DLPFC

**Figure 8:**
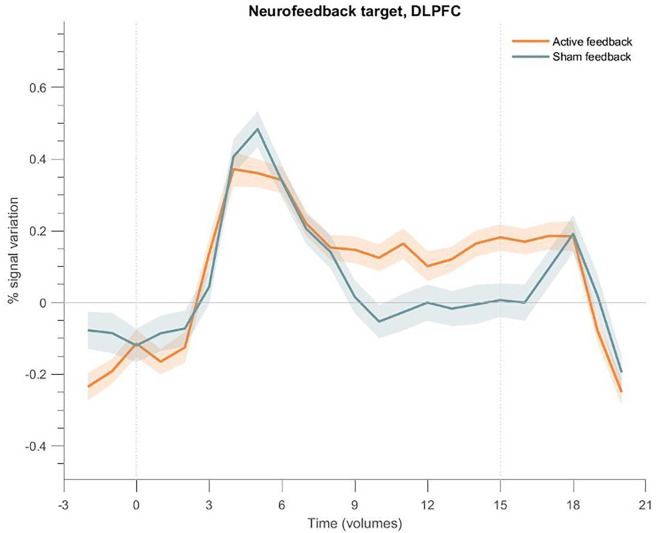
Event-related fMRI time courses considering the neurofeedback target region for the active neurofeedback group (orange line) and the Sham feedback group (blue line); the shaded regions correspond to the standard error of the mean (SEM). The first dotted vertical lines indicate the up-regulation block onset and the second to the end. DLPFC exhibits greater activation for the active feedback and sham feedback groups. Both groups present an increase at the beginning of the block, but only the active neurofeedback group sustains high activity levels; the Sham feedback group returns to baseline levels after a few seconds.

To check for differences in the BOLD signal response of the target ROI, we plot the event-related averages for the active and sham neurofeedback groups in Figure 8. Both groups show an activation peak at the beginning, but only the active neurofeedback group was able to sustain high activity levels until the end of the block.

#### Ventral striatum

**Figure 9:**
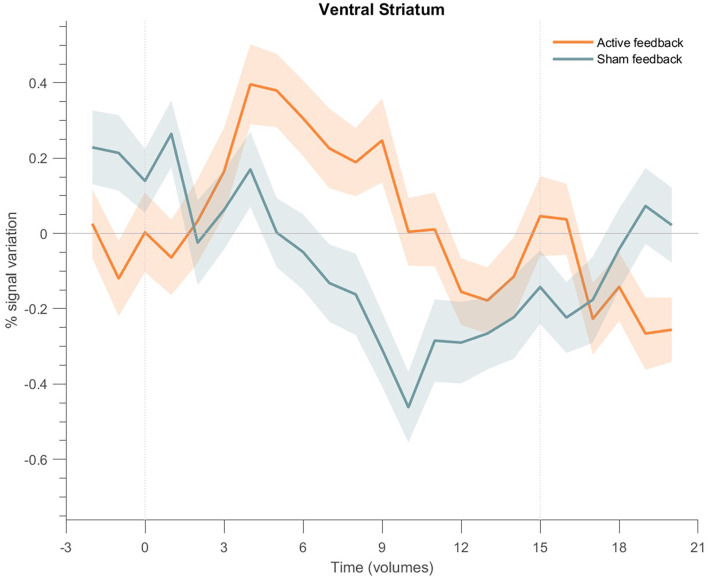
Event-related fMRI time courses considering the cluster on the ventral striatum for the active neurofeedback group (orange line) and the Sham feedback group (green line); the shaded regions correspond to the standard error of the mean (SEM). The first dotted vertical lines indicate the up-regulation block onset and the second to the end. The time courses show a positive transient response for the active neurofeedback group during the up-regulation condition, particularly at the beginning of each block, followed by the return to baseline activation values; on the opposite, the results show a negative response for the Sham group.

The same analysis was applied to the cluster on the ventral striatum (Figure 9), showing a PSC increase at the beginning of each block for the active neurofeedback group, returning to basal levels at the end of the block, and a negative response for the sham neurofeedback group.

### Debriefing

In the debriefing questionnaire, all the subjects in the active neurofeedback group perceived a correspondence between the given feedback and the imagery task. Some of these felt that this correspondence was independent of the strategies they used. Most participants followed the suggested imagery task for activating the DLPFC (inverted recall of self-generated numeric sequences), although some reported to rely more on sequence visualization and others on mental calculation. The reported number of digits varied from 4 to 20 and the number of sequences from 2 to 12, per block.

In the sham neurofeedback group, eight subjects (80%) reported no apparent association between feedback and imagery tasks. These participants tried different strategies, such as recalling numbers in a different language, repeating backwards the name of family members, and mentally playing an instrument. The reported number of digits varied from 3 to 16 and the number of sequences from 1 to 15, per block.

The number of digits and sequences did not differ significantly between groups (independent sample *t*-test, *p* = 0.496 for digits and *p* = 0.784 for sequences).

A table with participants’ answers to the debriefing questionnaire is provided as Supplementary Table 2.

## Discussion

In this study, we assessed the feasibility of self-regulating the activity of the DLPFC using a backward reciting digit imagery task and explored the neural underpinnings of working memory imagery and neurofeedback itself.

### Left lateralized working memory network is elicited by the imagery task

The group activation maps showed the expected recruitment of regions of the FPN (including DLPFC and PPC), both during the localizer and imagery runs. When comparing these two tasks, the imagery results are left lateralized, as confirmed by the calculated laterality index (0, 26). This is consistent with the previous knowledge regarding the neural correlates of verbal working memory tasks. N-back task (localizer) elicits bilateral activation of lateral pre-frontal and parietal regions with lateralization possibly varying according to age and task difficulty (Owen et al., 2005; Rottschy et al., 2012; Emch et al., 2019). Backwards reciting task has a correspondent left lateralized functional map (Smith et al., 1996), regardless if the self-generated sequence is numerical or alphabetical, and with backward recitation requiring additional neural resources than forward recitation, mainly in parietal areas (Zhou et al., 2006), both used in our paradigm.

Additionally, functional maps showed (1) positive clusters in basal ganglia and thalamus, also known to be involved in working memory (Lewis et al., 2004); and (2) negative clusters in DMN representative of the well-known anti-synergic coupling (with negative correlation) of FPN-DMN. Both the interaction between FPN and DMN and the cortico-subcortical connectivity has already been shown, by Zhang’s group, to be modified by rt-fMRI working memory neurofeedback, with consequent performance modulation on post-training (Shen et al., 2015; Zhang G. et al., 2015, Zhang Q. et al., 2015; Zhang et al., 2016).

### Active NF group has higher and more sustained signal modulation of DLPFC

All participants from the active group and the majority from the sham group were able to increase DLPFC activity already in the initial training, before neurofeedback runs, indicating that the proposed imagery task, in a population without executive dysfunctions, is robust enough to recruit and maintain DLPFC activity without any type of feedback.

However, we also expected that the active NF group would be able to achieve greater control over signal modulation in the DLPFC when compared to the sham-control group, given the correspondence between feedback and mental strategy (Sorger et al., 2019). Indeed, our results show a significant difference in DLPFC activity during the neurofeedback runs, being higher in the active NF group. On the contrary, such differences were not found during the runs without feedback (train and transfer), denoting that closing the neurofeedback loop with valuable information increases the mean target ROI activation, a result that is consistent with improved self-modulation ability. Behaviorally, this was also implicit in the fact that only participants from the sham group tried different imagery strategies, while in the active group all maintained the provided suggestions, despite equal instructions for both groups. This is a reflection of the associative learning framework supporting neurofeedback, since reinforcement of the used mental strategy occurs when feedback acts as a reward.

In addition to previous neurofeedback studies engaging DLPFC, we demonstrate that active and sham neurofeedback groups differ not only in the modulation amplitude but also in the way DLPFC activity progresses along the NF run. Active neurofeedback participants were able to sustain higher levels of BOLD activity along all the run, while in the sham group it drops to null values after nine volumes from the imagination task onset. Again, this is probably due to the acknowledgement, as the run proceeds, of the non-contingency between their effort and the represented signal change.

Another study using DLPFC as a neurofeedback target, found differences in the dynamical regulation of physiological DLPFC activity after neurofeedback training, although without a modification of target activation levels (van den Boom et al., 2018). In this case, there was a reduction in time needed to return to baseline, suggesting that is possible to deactivate DLPFC in a deliberate way. They also stated that control of the elevation phase is more difficult to achieve with the neurofeedback since the execution of a WM task immediately increases to maximum the BOLD signal. In our data, during feedback runs, we also find an activity peak approximately at 6–8 s both for active and sham groups and the main difference arises after, on the ability to sustain it and not returning to baseline. However, van den Boom et al. (2018) only presented their data on the post-test, so we cannot compare the dynamics of DLPFC BOLD activity during feedback from this study with our own. Another important difference, since we are arguing on the contingency relevance, is that in their study sham group received feedback from another participant, while in our protocol feedback it was derived from white matter voxels.

### Motivation may be the key factor differentiating group performance

In line with our hypothesis that was mainly the motivation differentiating both groups, when we performed an exploratory whole-brain analysis we found a single cluster in the ventral striatum (in particular, nucleus accumbens) with higher activation in NF group compared to sham group on NF runs. The ventral striatum has been pointed as responsible for unconscious reward processing in NF, with anterior cingulate cortex and anterior insular cortex being involved in the conscious counterpart of reward and NF perception, as reviewed by Sitaram et al. (2017). The nucleus accumbens, in particular, is recognized as key integrative region of both motivational and learning-memory circuits, receiving input from cortical areas (including DLPFC), limbic system, and midbrain (substantia nigra/ventral tegmental area) and, in turn, selecting appropriate responses (Camara et al., 2009).

The event-related time course analysis performed on this cluster during NF runs showed a positive response at the beginning of each block for the active NF group contrasting with a negative initial response and during almost all the block for the sham group during NF runs. In the active NF group, the BOLD signal follows the predicted pattern, peaking at approximately 6 s and then slowly returning to baseline, according to previous reports that nucleus accumbens activity is time-limited (i.e., not sustained) by dopamine metabolism (Knutson and Gibbs, 2007; Greer et al., 2014). In the sham group, although there is an initial overshooting (lower than the active group), it is followed by a negative response all along the block. Interestingly, this relationship inverts on down-regulation (baseline), where participants in the sham group may percept an apparent causality between their effort to zero the thermometer and a BOLD sign that actually does not represent cortical activity (with lower amplitude). When neurofeedback is taken from the equation (train and transfer runs) the difference between groups on time course analysis dissolves, as expected. Furthermore, subjectively, all the participants in the active group perceived a correlation between feedback and performance—against only 20% of the sham group, with subjects reporting frustration along NF runs.

Conversely, striatal and midbrain structures included in the reward network have been tested as the direct target in neurofeedback experiments, searching for therapeutic effects on diseases affecting the mesolimbic dopamine system. These studies offer a mirrored perspective which complements our results, showing how these structures contribute to successful learning in neurofeedback and how they are linked to other brain regions. The experiment of MacInnes et al. (2016) is relevant to understanding temporal BOLD signal dynamics during neurofeedback and how directly targeting the mesolimbic dopamine system contributes to learning volitional cognitive strategies. They also found sustained activation in the target region (ventral tegmental area—VTA) during all the 20 s of the trial as a proof of improvement, but which was only present in the post-test run, reflecting a learning effect (which was not present in our study). During neurofeedback runs (compared to pre-test) they showed higher connectivity VTA-caudate, VTA-hippocampus and NAcc-caudate, also emphasizing the role of NAcc in neurofeedback training targeting the reward network. A recent article (Hellrung et al., 2022) similarly targeting VTA/SN provides a more profound investigation of which specific neural mechanisms are related to the transfer success when training VTA, showing that the most successful individuals had stronger activation of cognitive control areas, mainly the prefrontal cortex, during transfer. That is, higher individual reward-related sensitivity in the DLPFC increases the chance of neurofeedback training success. Links between these networks are bidirectional and it has been proved that dopamine action in DLPFC sustains working memory performance (Arnsten et al., 2015). In conclusion, associative learning crucially contributes to real-time fMRI neurofeedback effects, as also suggested by our results.

### Implications to advances in the RT-fMRI neurofeedback field and future directions

Previous neurofeedback studies targeting DLPFC for working memory enhancement (Zhang et al., 2013; Sherwood et al., 2016a,b; van den Boom et al., 2018), and also our own, show that DLPFC readily activates with the explicitly suggested imagery task, with improved self-modulation ability provided by neurofeedback. However, in contrast to our study, none of the previous studies investigate motivational effects or the participant perception of feedback contingency and how it may influence NF outcomes. Directly linked to this issue, the choice of an adequate control condition is being highly debated in the neurofeedback community and also varied across studies. It is suggested that an ideal control must minimize the likelihood of placebo effects, while maintaining neurophysiological specificity and promote equal motivation/perception of success (Sorger et al., 2019). However, it is particularly challenging when targeting such a key core network and hub (as the executive network and in particular the DLPFC, which are efficiently activated by a working memory imagery task and by neurofeedback processing itself), to find a control ROI regulated as easily, with similar signal properties, while not being related to it. That is, both the sensitivity and the specificity of the control region are key aspects to consider (Sorger et al., 2019).

In our study, we established a link between motivational aspects and DLPFC self-modulation through rt-fMRI neurofeedback. Notably, although sham participants could not identify a correspondence between used strategies and given feedback, all except one were able to provide an answer to the last two questions of the debriefing, discriminating better and worse strategies. Also, the number of digits and sequences did not differ significantly between groups, suggesting that both were equally engaged on the imagery task. Another critical point is that none of the participants was aware of a control condition, that is, they did not know that receiving false feedback was a possibility in this type of experiment (as they were naïve to neurofeedback experiments). In this sense, our results suggest that the perception of non-contingency did not affect effort, perseverance on the task, or the belief that the feedback was real, but had a motivational impact, which is supported by fMRI data.

Perception of contingency is vital for the operant conditioning that is a foundational principle of neurofeedback and we find motivational aspects difficult to be removed from the equation, at least in neurofeedback tasks targeting executive functions. Critically, they must be considered when reporting and interpreting neurofeedback results, having implications on possible adjustments to achieve optimized experimental design and, ultimately, in translation to clinical implementation.

### Limitations

We were not able to prove a learning effect from our neurofeedback training, with both groups equally performing in the transfer run. This was anticipated when we designed a single-session neurofeedback experiment, being our main goal to study the general capacity of individuals to regulate DLPFC (feasibility test). Although the optimal number of neurofeedback sessions is not yet established in the literature (possibly varying according to target and population), it is consensual that a single session will hardly induce changes in neural processes (Thibault et al., 2018; Paret et al., 2019; Fede et al., 2020). The fact that our participants were already able to modulate DLPFC activity in train run, alludes to a possible initial ceiling effect, precluding detection of within session learning and perhaps contributing to justify the equal performance on transfer. Furthermore, we did not control for fatigue or habituation, which may also interfere in the results of the final run.

Another limitation is that our paradigm was not specifically designed to unscramble task-dependent DLPFC BOLD-magnitude from the engagement on neurofeedback process itself, namely in the preparation and execution of mental strategies (Skottnik et al., 2019). Sherwood et al. (2016b) showed that left DLPFC activity evolves interchangeably in closed and open-loop neuromodulation across training. However, we argue that the cognitive control globally linked to neurofeedback training is equally present for sham feedback, as previously reported by Ninaus et al. (2013), so that the differences we found in DLPFC were related to the imagery task.

Here, we did not correct for physiological artifacts in feedback signal computation. As far as we know, there is no gold standard for the use of derivatives of cardiac and respiratory signals as confounds in real-time. However, the impact of these regressors has been studied in recent experiments performing connectivity-based neurofeedback (Weiss et al., 2020). With similar objectives, the use of a control region has been applied to account for global fluctuations (Dewiputri and Auer, 2013).

Finally, our results on reward network during neurofeedback training derived from a whole-brain exploratory analysis, demanding further corroboration in a larger sample and with different target areas. Here, we did not present a sampling plan. Since our study is a proof-of-concept, the sample size rationale was based on the literature of previous NF studies targeting DLPFC for executive functions enhancement (Zhang et al., 2013; Sherwood et al., 2016a,b; van den Boom et al., 2018).

### Future perspectives

The present results suggest that neurofeedback promotes not only greater signal modulation of DLPFC, but also a more sustained activity of this target region along the neurofeedback runs. This ability seems associated with motivation, since: (1) differences dissolved when neurofeedback is removed; and (2) when comparing functional maps from active NF and sham group, we found a single cluster located on the ventral striatum, responsible for unconscious reward processing.

DLPFC was easily activated by all subjects in most of the runs (with or without feedback) and is clearly modulated by neurofeedback, with the critical involvement of the reward network. It is also a large and superficial brain area, easily accessible by other technics (such as EEG, MEG or fNIRS), which makes it an ideal target for training transfer. Its clinical potential is reinforced by the important role that DLPFC plays in many high-level cognitive functions. Thus, alterations to its normative functioning are linked to the cognitive impairment found in a number of neuropsychiatric disorders, which ultimately largely impacts individual functional capacity and independence. Taking all together, DLPFC emerges as a clinically-relevant neurorehabilitation and/or neuroenhancement target, that deserves future translational research in the neurofeedback field.

## Data availability statement

The raw data supporting the conclusions of this article will be made available by the authors, without undue reservation.

## Ethics statement

The studies involving human participants were reviewed and approved by Comissão de Ética da Faculdade de Medicina da Universidade de Coimbra. The patients/participants provided their written informed consent to participate in this study.

## Author contributions

DP and MC-B conceived the study. DP, AS, JP, BD, and MC-B designed the study. DP and SM performed the recruitment. DP, BD, AS, and JP acquired and analyzed the data. DP, AS, JP, SM, AM, BD, and MC-B discussed the results. DP wrote the manuscript with contributions from AS, JP, and BD. MC-B reviewed the manuscript. All authors contributed to the article and approved the submitted version.
