# Supplementary material for: Neurofeedback-dependent influence of the ventral striatum using a working memory paradigm targeting the dorsolateral prefrontal cortex
Source: Front Behav Neurosci. 2023 Feb 9;17:1014223. doi: 10.3389/fnbeh.2023.1014223 (PMC9947361; doi:10.3389/fnbeh.2023.1014223)
Supplement: Supplementary file 1 [file Table_1.DOCX]

| **Table 1 - Summary of selected ROIs in the DLPFC for the active NF (a) and sham group (s) - coordinates of the center in Talairach space, the number of voxels, and t-value for the contrast ‘2-back’>Baseline.** | | | | | |
| --- | --- | --- | --- | --- | --- |
| **Participant** | **x** | **y** | **z** | **NrOfVoxels** | **t value** |
| **a1** | -41,32 | 7,41 | 41,56 | 4972 | 5,77 |
| **a2** | -29,53 | 31,69 | 37,57 | 3966 | 8,82 |
| **a3** | -41,16 | 30,8 | 21,86 | 3571 | 6,35 |
| **a4** | -42,88 | 20,11 | 41,19 | 3134 | 6,82 |
| **a5** | -37,64 | 6,55 | 38,52 | 3556 | 5,02 |
| **a6** | -39,28 | 34,25 | 42,04 | 4720 | 5,69 |
| **a7** | -39,19 | 31 | 40,21 | 4110 | 11,00 |
| **a8** | -45,79 | -1,73 | 44,72 | 1741 | 9,88 |
| **a9** | -40,78 | 20,52 | 36,01 | 2184 | 10,73 |
| **a10** | -40,39 | 16,01 | 40,46 | 5669 | 7,84 |
| **a11** | -41,2 | 12,25 | 34,78 | 5487 | 5,91 |
| **a12** | -43,48 | 26,21 | 33,25 | 2940 | 8,56 |
| **a13** | -44,43 | 29,04 | 36,07 | 766 | 12,94 |
| **a14** | -37,78 | 15,18 | 36,87 | 2595 | 7,90 |
| **a15** | -46,1 | 14,03 | 28,63 | 2241 | 8,82 |
| **a16** | -32,48 | 31,15 | 33,57 | 2763 | 10,48 |
| **a17** | -42,92 | 24,98 | 36,22 | 2457 | 8,91 |
| **s1** | -40,88 | -1,61 | 41,63 | 798 | 5,12 |
| **s2** | -37,65 | 14,19 | 25,68 | 1974 | 6,75 |
| **s3** | -38,55 | 27,37 | 40,65 | 1455 | 7,24 |
| **s4** | -44,19 | 18,11 | 35,69 | 1201 | 5,07 |
| **s5** | -43,31 | 17,08 | 43,17 | 969 | 8,96 |
| **s6** | -48,56 | 9,13 | 28,76 | 1096 | 8,68 |
| **s7** | -41,13 | 19,6 | 38,42 | 1312 | 3,86 |
| **s8** | -45,45 | 26,58 | 31,94 | 841 | 7,29 |
| **s9** | -50,22 | 15,46 | 28,21 | 1208 | 6,48 |
| **s10** | -44,6 | 18,22 | 35,82 | 1215 | 5,25 |
| Mean | -41,51 | 19,02 | 36,06 | 2553,37 | 7,63 |
