# Supplementary material for: Neurofeedback-dependent influence of the ventral striatum using a working memory paradigm targeting the dorsolateral prefrontal cortex
Source: Front Behav Neurosci. 2023 Feb 9;17:1014223. doi: 10.3389/fnbeh.2023.1014223 (PMC9947361; doi:10.3389/fnbeh.2023.1014223)
Supplement: Supplementary file 2 [file Table_2.DOCX]

| **Table 2. Answers to Debriefing Questionnaire** | | | | | |
| --- | --- | --- | --- | --- | --- |
| **Subject** | **1** | **2** | **3** | **4** | **5** |
| **Sham group** | | | | | |
| 1 | Well, although some discomfort in the legs | No | maximum number of sequences 12; maximum number of digits: 16 | Counting even numbers in Italian, until 21 and backwards. Count odd numbers until 10 and backwards | Multiplications, subtractions, multiples of a specific number, sequential count |
| 2 | I felt confident during the first task without feedback and I was able to distinguish what was asked in baseline and imagination. Posteriorly, I felt difficulty in increasing the thermometer levels | Yes, although with some delay | mean of 10 sequences; maximum number of digits: 4 | Sequences with 4 digits corresponding to subtractions | When I tried to abstract by fixating a point on the screen or the word “baseline”, sometimes I raised the thermometer |
| 3 | Very anxious, since this was the first time on an MRI scanner | Yes | 2 to 3 sequences; 10 to 12 digits | Bigger numbers (200, 300), two by two | Simpler sequences, like 1,2, 3, 4, 5… |
| 4 | Calm, comfortable, well | no | 5+5 | none | all |
| 5 | Frustrated with lack of feedback, but performed the session with tranquillity and with no difficulties | I could not find any strategy that has resulted in a appropriate feedback | 7 sequences, 15 digits | No relation with feedback | No relation with feedback |
| 6 | It was difficult to control the basal effort. I felt tired with the repetitions of the exercises. Felt I could not find any more exercises to stimulate my thoughts | Not always. Many times, I thought I was making a bigger effort and there was no correspondence. And when it went to baseline, the level increased | Don’t remember | Sequences with bigger numbers (more than 30), Say backwards full names of family members | Repeat a lot the sequences |
| 7 | I felt that sometimes my strategies did not work, but I felt ok | Feedback was not very high, but I could see the delay between feedback and baseline | Usually, I tried to imagine one or two sequences per block and increase it as far as possible. Maybe 10 digits | I couldn’t see a relationship between sequences complexity and feedback. When I squint feedback level seemed to increase… | It was hard to understand how to increase feedback. |
| 8 | Not bad | I tend to answer no. | 3, 3 | When I tried to imagine playing the musical instrument I play, not only in spatial terms, but timber and tone | Simple number sequences, mental calculation |
| 9 | Normal | Rarely | 15 sequences, 9 digits | Number repetition | Imagination and baseline |
| 10 | Well, but not very focus due to the noise and anxiety | It seemed there was no correspondence even when I made an effort to imagine a sequence that make me think more | 3 sequences, 10 digits per sequence | When I used methods that make me think more and that needed more focus | Those simpler sequences, that became repetitive and didn’t need a greater focus |
| **Neurofeedback group*** | | | | | |
| 1 | ok | I felt differences between imagination blocks and baseline, but quite independent on strategies | 4-6 sequences, maximum 6 digits | sequences of 5 non-consecutive numbers | none |
| 2 | Comfortable until the moment the headphones dislocated slightly, making it difficult to concentrate on the task, but it was more at end of the session | yes and it get significantly better across the experience | 5 sequences, 20 digits | known sequences which familiarity allowed to invert the direction easily | trying to increase the difficulty of the sequence, increasing the interval between digits, because when inverting the effort is bigger and attention shifts to calculation |
| 3 | unfocused on transer run promoted by phone noises | yes in most of the used strategies for both activation and baseline runs | 9/10 sequences with 4/5 digits or 4/5 sequences with 9 digits | bigger numbers, building bigger number sequentially (5 digits and then add until 9) | numbers with less digits |
| 4 | Feedback sometimes get me distracted, there was a delay between strategy and result | yes | 10 sequences, 6 digits | visualization of numbers in screen, partially ignore feedback | concentrate too much on feedback |
| 5 | well | yes | 2 sequences, 8 digits | bigger sequences, 2 or more digits in each sequence |  |
| 6 | ok | yes, mainly with repetition if feedback runs | 3 or 4 sequences, 6 digits | generate sequences of numbers including more than 2 digits and non-monotonic, that is that not increase or decrease from beginning to end | sequences starting in 1 or with other small numbers and sequences associated to something or someone. |
| 7 | it was tiring | yes | 10-12 sequences, 7 digits | visualize numbers | sequential numbers |
| 8 | nothing in particular, some anxiety when I couldn't make the thermometer going up or down as I wanted | yes | 4/5 sequences per block with 4/5 numbers | visualize in imagination a number sequence and trace the ascending and descending pathway | imagine numbers without visual reference |
| 9 | very lonely | yes | 4 sequences, 10 digits | sequences with numbers in a row | sequences with aleatory numbers |
| 10 | normal (quite sleepy) | sometimes, but in general yes | 4 sequences, 7 digits | invert number sequence that are already know (phone numbers, birthdays). Associate each square of the thermometer to a number and invert order | invert sequences of aleatory numbers, without associate each digit to something. It went difficult for sequences with more than 4/5 digits. |
| 11 | very well | yes | 4 to 5 sequences, 5 to 8 | sequences of 15 digits imagining very quickly | think slow |
| 12 | I felt that was never really able to control feedback | I felt few correspondence between strategy and feedback. | 4 numbers, 4-6 sequences | binary sequences, sequences with repeated numbers | pre-defined sequences |

**Questions:**

1. How did you feel during neurofeedback session?
2. Did you find a correspondence between used strategies and given feedback?
3. Which was the maximum number of sequences you could picture in each block? And maximum number of digits per sequence?
4. Which strategies worked better?
5. Which strategies did not work?

* Missing data from 5 participants in the neurofeedback group is due to the fact that in the beginning of this research project we were only performing oral debriefing.
