# Supplementary material for: Neurofeedback-dependent influence of the ventral striatum using a working memory paradigm targeting the dorsolateral prefrontal cortex
Source: Front Behav Neurosci. 2023 Feb 9;17:1014223. doi: 10.3389/fnbeh.2023.1014223 (PMC9947361; doi:10.3389/fnbeh.2023.1014223)

TASK INSTRUCTIONS FOR PARTICIPANTS

LOCALIZER

The first task you will have to complete inside the MRI machine is called “n-back” and your attention is required in order to complete it successfully.

We will provide a device with a button for you to press according to these instructions.

When there is a cross on screen, you should look at it and relax. The rest of the time, you will be shown a random sequence of numbers and you should do one of two tasks:

1) The 1-back task, will be instructed as “recall one number before”. This means you should press the button whenever a number is repeated. For example, in the sequence “2 3 4 5 5 6 7 8 9 8 1 4 4 2 4”, the underlined numbers would be the ones signaled by pressing the button.
2) The 2-back task is harder than the previous one and will be instructed as “recall two numbers before”. This means that you should press the button when the number displayed is the same as two digits before. For example, in the sequence 2 3 4 5 6 5 7 8 9 8 1 3 4 2 4, the underlined numbers would be the ones signaled by pressing the button.

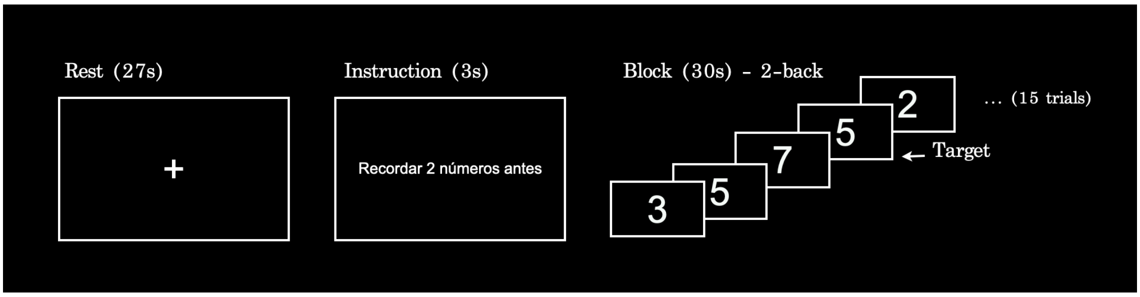

NEUROFEEDBACK PARADIGM

The goal of the second tier of the MR study we will perform is to regulate your own brain activity according to the feedback you will be given. During part of the study, we will display a locking cross on a black background. This is when you should be as relaxed as possible, without any mental effort. When a thermometer is displayed, you should begin this task. This thermometer is your feedback, and the bars will increase or decrease according to the activation level. What we intend is for you to think of a numeric sequence and then repeat it backwards without verbalizing. For example, “237” > “732”. Repeat this task as long as there is a thermometer on screen, and try to achieve as high a level as you can. You may use any digits as you wish. You can change the difficulty of your own task in the way you find it to be more effective. For example, you can increase the sequence’s length, the variability (for example, 2525 will be easier than 3794), the speed at which you try reverse the sequence or any other strategy you find effective.

In the end, we will ask you what strategies you used.

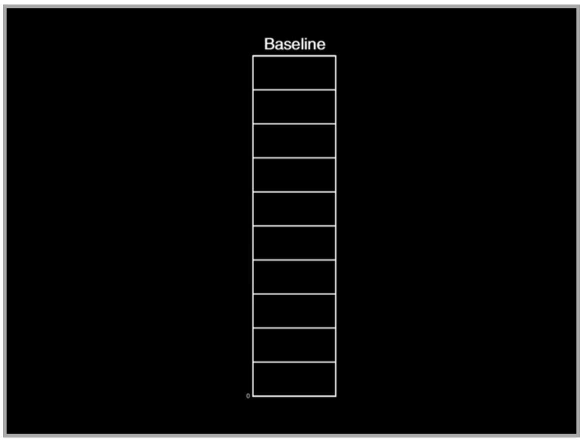

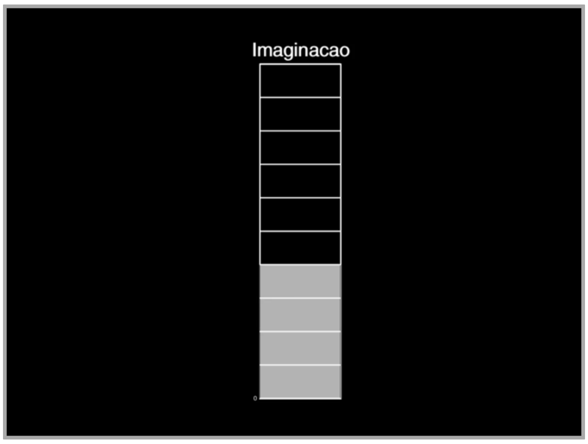

Supplement: Supplementary file 3 [file Data_Sheet_1.PDF]
