# Supplementary material for: Neurofeedback-dependent influence of the ventral striatum using a working memory paradigm targeting the dorsolateral prefrontal cortex
Source: Front Behav Neurosci. 2023 Feb 9;17:1014223. doi: 10.3389/fnbeh.2023.1014223 (PMC9947361; doi:10.3389/fnbeh.2023.1014223)
Supplement: Supplementary file 4 [file Data_Sheet_2.PDF]

CRED-nf checklist summary

20 December, 2022

Manuscript title: Neurofeedback-dependent influence of the ventral striatum using a working memory paradigm targeting the dorsolateral prefrontal
Corresponding Author: Daniela Jardim Pereira
Corresponding author email: danielajardimpereira@gmail.com

Item No.	Checklist item	Manuscript Details
**Pre-experiment**		
1a	Pre-register experimental protocol and planned analyses	*This experiment was not preregistered*
1b	Justify sample size	Since our study is a proof-of-concept, the sample size rational was based on the literature of previous NF studies targeting DLPFC for executive functions enhancement (Zhang et al., 2013; Sherwood et al., 2016a, 2016b; van den Boom et al., 2018)
**Control groups**		
2a	Employ control group(s) or control condition(s)	Seventeen subjects were allocated to the active neurofeedback group (11 male, mean age 27.8 +/- 4.2 years) and ten subjects to the sham neurofeedback group (5 male, mean age 26.2 +/- 2.9 years). (...) For the sham feedback group, we selected between 18 and 24 functional voxels of white matter, evenly distributed per hemisphere, in each participant centrum semiovale.
2b	When leveraging experimental designs where a double-blind is possible, use a double-blind	*NA: A double-blind was not appropriate for this experiment*
2c	Blind those who rate the outcomes	*Those who rated the outcome were not blind to group assignment*
	Blind those who analyse the data	*Those who analysed the data were not blind to group assignment*
2d	Examine to what extent participants and experimenters remain blinded	After the scanning session, participants answered a debriefing questionnaire that included subjective questions about their feelings during the acquisition (How did you feel during the NF session?), the contingency between effort and feedback change (Did you feel there was a correspondence between the used strategies and the given feedback?) and the strategies they used (What was the maximum number of sequences you could picture in each block? And maximum digit number? Which strategies worked better? And which ones did not work?).

2e	In clinical efficacy studies, employ a standard-of-care intervention group as a benchmark for improvement	*NA: This is not a clinical efficacy study*
**Control measures**		
3a	Collect data on psychosocial factors	*Psychosocial factors were not measured*
3b	Report whether participants were provided with a strategy	Subjects were instructed to use a cognitive strategy of backward reciting the self-generated sequences sub-vocally to increase the number of bars in the thermometer (Zhang et al., 2013). The content, length, and difficulty of the sequences they generated and the speed of recitation could be adjusted according to the feedback.
3c	Report the strategies participants used	In the debriefing questionnaire, all the subjects in the active neurofeedback group perceived a correspondence between the given feedback and the imagery task. Some of these felt that this correspondence was independent of the strategies they used. Most participants followed the suggested imagery task for activating the DLPFC (inverted recall of self-generated numeric sequences), although some reported to rely more on sequence visualization and others on mental calculation. The reported number of digits varied from 4 to 20 and the number of sequences from 2 to 12, per block. In the sham neurofeedback group, eight subjects (80%) reported no apparent association between feedback and imagery tasks. These participants tried different strategies, such as recalling numbers in a different language, repeating backwards the name of family members, and mentally playing an instrument. The reported number of digits varied from 3 to 16 and the number of sequences from 1 to 15, per block. The number of digits and sequences did not differ significantly between groups (independent sample t-test, p=0.496 for digits and p=0.784 for sequences). (Complete answers to the debriefing in supplementary material)
3d	Report methods used for online-data processing and artifact correction	(for online-data processing, but not for artifact correction) For online ROI definition, we functionally targeted DLPFC using the real-time fMRI software package Turbo-BrainVoyager 3.2 (TBV; Brain Innovation, Maastricht, The Netherlands). Real-time preprocessing included 3D head motion correction (6 degrees of freedom) compared to first volume. Online statistical analysis of incoming volumes was incremental, using a recursive least squares general linear model (GLM) based on a design matrix automatically created from the imported stimulation protocol and including the convolution of the BOLD time course with a two-gamma hemodynamic response curve (HRF).
3e	Report condition and group effects for artifacts	*Condition and group effects for artifacts were not measured, or not reported in the manuscript*

4a	Report how the online-feature extraction was defined	Activation clusters were estimated, in a first approach, according to the contrast '2-back'>'baseline' that usually resulted in the highest percent of signal change (PSC). However, in some participants, we found very large clusters of activation in DLPFC (merging with pre-motor areas) with this contrast. In these cases, switching to the '2-back'>'1-back´ contrast allowed us to delineate a more circumscribed effort-related cluster, related with cognitive-load and manipulation, and find a more accurate peak voxel to center the rectangle drew on multi-slice view of TBV. The defined rectangle extended to the slice above and below (total of 3 slices) and the average of significant voxels was displayed on the time-course. We generally considered ROIs appropriate as NF targets, when its PSC was around 1% or more. Anatomical references were also taken into account by an expert neuroradiologist (DJP) to determine DLPFC, guaranteeing it was located anterior to the premotor cortex and superior to the planes including the lateral ventricles. All targets were selected on the left hemisphere since participants were performing a verbal working memory task during imagery runs (Emch et al., 2019). For the sham feedback group, we selected between 18 and 24 functional voxels of white matter, evenly distributed per hemisphere,
		in each participant centrum semiovale.
4b	Report and justify the reinforcement schedule	In this sham-controlled single session study, we validate a rt-fMRI neurofeedback training framework for DLPFC self-modulation using a working memory paradigm on healthy subjects (without executive dysfunction).
4c	Report the feedback modality and content	During the neurofeedback runs, apart from the first baseline block, visual feedback was provided in the form of a thermometer that was updated every TR based on the mean ROI activation of the neurofeedback target selected during the localizer run
4d	Collect and report all brain activity variable(s) and/or contrasts used for feedback, as displayed to experimental participants	The thermometer was divided into 10 discrete levels with a maximum value of 2.5%, where each level represented a given range of percent BOLD signal change (0 for an empty thermometer and 0.25% for each level). The feedback value fb for the current time point n is calculated within each block given the current value val, a baseline level bl (mean BOLD value in the target region, during previous 'baseline' block) according to equation 1:The thermometer was divided in 10 levels, where each represented a given percent BOLD signal change (PSC), compared with the previous 'Baseline' block average value, considering a maximum value of 2.5 (Equation 1).<br>fb(n)=(valx(n)-bl)/bl×100 (1)
4e	Report the hardware and software used	For online ROI definition, we functionally targeted DLPFC using the real-time fMRI software package Turbo-BrainVoyager 3.2 (TBV; Brain Innovation, Maastricht, The Netherlands).
**Outcome measures - brain**		

5a	Report neurofeedback regulation success based on the feedback signal	First, we computed the t-value in DLPFC for the contrast of interest ('imagery'>'baseline') for all imagery runs in both groups. We used this value as the measure of the participant's ability to modulate the target region.
5b	Plot within-session and between-session regulation blocks of feedback variable(s), as well as pre-to-post resting baselines or contrasts	(single session, yes for pre-test, post-test No significant differences were found on paired sample t-test between train and transfer runs on the active NF group (Figure 4) or in sham group (no learning effect).
5c	Statistically compare the experimental condition/group to the control condition(s)/group(s) (not only each group to baseline measures)	We did not find a significant interaction between group assignment and runs (F(4,22)=1.617), p=0.205) in the mixed-model ANOVA. However, we found a significant effect for group assignment (between-subject) (F(1,77)=5.056, p=0.034). Within neurofeedback runs, post-hoc independent sample t-tests (with Bonferroni correction) show significant differences of DLPFC activity between groups in runs 1 and 3, as represented on Figure 3. When comparing runs with feedback and without feedback (train and transfer) to evaluate group differences related to feedback contingency (two-way ANOVA), we found a group assignment effect (F(2,24)=3.860, p=0.035) with significant difference between groups only in neurofeedback runs (p=0.014) on post-hoc pairwise comparisons (with Bonferroni correction).
**Outcome measures - behaviour**		
6a	Include measures of clinical or behavioural significance, defined a priori, and describe whether they were reached	*NA: the study does not take cognitive or behavioural measures*
6b	Run correlational analyses between regulation success and behavioural outcomes	*NA: the study does not take cognitive or behavioural measures*
**Data storage**		
7a	Upload all materials, analysis scripts, code, and raw data used for analyses, as well as final values, to an open access data repository, when feasible	*No additional documents related to the materials, analysis scripts, code, raw data, or final values are available for this manuscript*
