# Supplementary material for: Neurofeedback-dependent influence of the ventral striatum using a working memory paradigm targeting the dorsolateral prefrontal cortex
Source: Front Behav Neurosci. 2023 Feb 9;17:1014223. doi: 10.3389/fnbeh.2023.1014223 (PMC9947361; doi:10.3389/fnbeh.2023.1014223)
Supplement: Supplementary file 5 [file Data_Sheet_3.PDF]

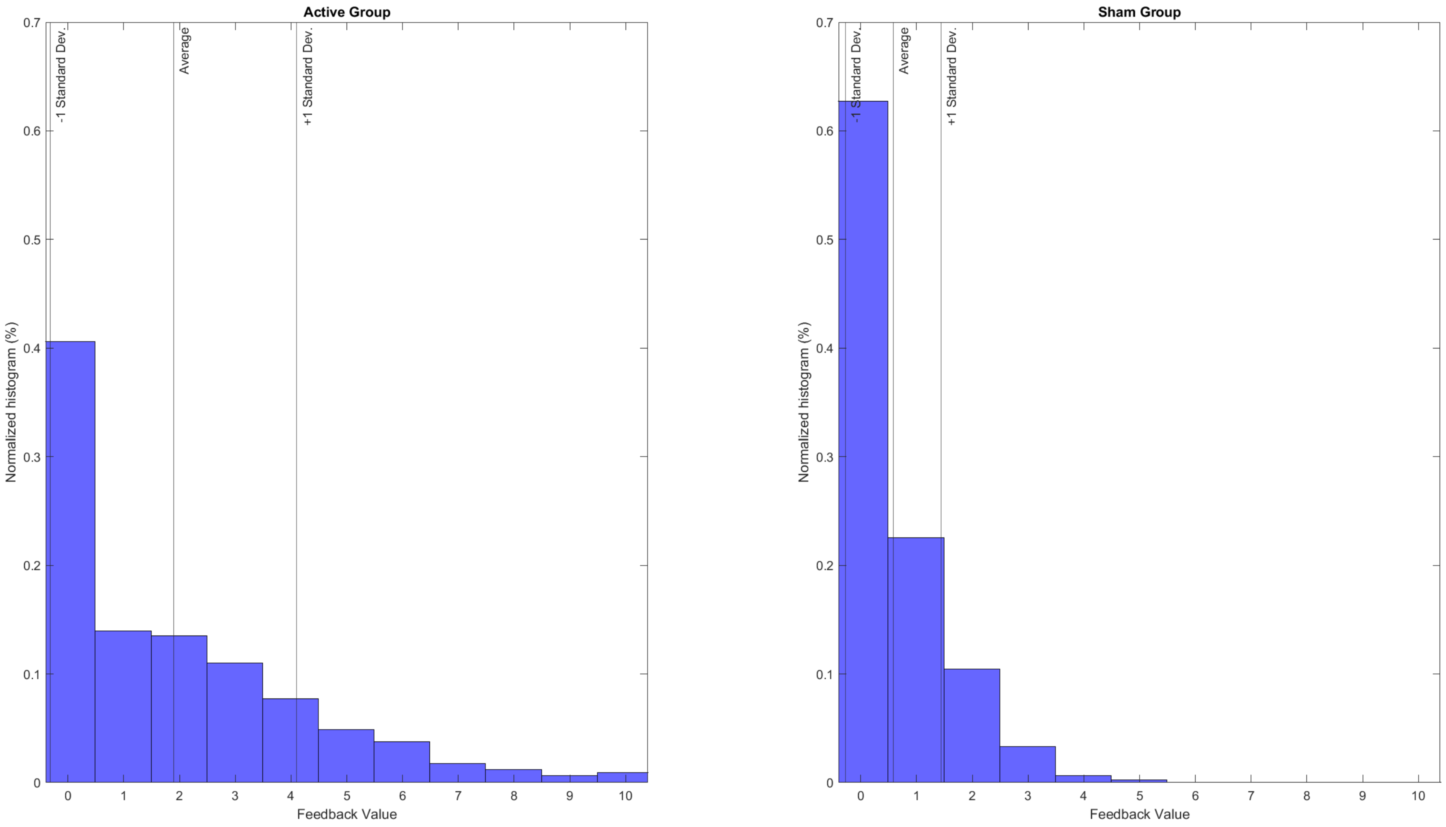

Figure S1 - Histograms for active (left) and sham (right) groups representing the distribution of *fb* (each time point) throughout the entire neurofeedback runs (all baseline and imagery blocks), by thermometer level (0 to 10) ($mean_{Active} = 1.89 \pm 2.21$; $mean_{Sham} = 0.58 \pm 0.85$).
